# Patient Experiences With Full Electronic Access to Health Records and Clinical Notes Through the My Health*e*Vet Personal Health Record Pilot: Qualitative Study

**DOI:** 10.2196/jmir.2356

**Published:** 2013-03-27

**Authors:** Susan S Woods, Erin Schwartz, Anais Tuepker, Nancy A Press, Kim M Nazi, Carolyn L Turvey, W. Paul Nichol

**Affiliations:** ^1^Portland VA Medical CenterHealth Services Research & DevelopmentPortland, ORUnited States; ^2^Oregon Health & Science UniversityDepartment of Medical Informatics & Clinical EpidemiologyPortland, ORUnited States; ^3^Schools of Nursing and MedicineOregon Health & Science UniversityPortland, ORUnited States; ^4^Veterans and Consumers Health Informatics OfficeOffice of Informatics & AnalyticsVeterans Health AdministrationWashington, DCUnited States; ^5^Center for Comprehensive Access & Delivery Research and EvaluationIowa City VA Health Care SystemIowa City, IAUnited States; ^6^VA Puget Sound Health Care SystemSeattle, WAUnited States

**Keywords:** personal health records, eHealth, patient access to records, veterans, patient participation

## Abstract

**Background:**

Full sharing of the electronic health record with patients has been identified as an important opportunity to engage patients in their health and health care. The My Health*e*Vet Pilot, the initial personal health record of the US Department of Veterans Affairs, allowed patients and their delegates to view and download content in their electronic health record, including clinical notes, laboratory tests, and imaging reports.

**Objective:**

A qualitative study with purposeful sampling sought to examine patients’ views and experiences with reading their health records, including their clinical notes, online.

**Methods:**

Five focus group sessions were conducted with patients and family members who enrolled in the My Health*e*Vet Pilot at the Portland Veterans Administration Medical Center, Oregon. A total of 30 patients enrolled in the My Health*e*Vet Pilot, and 6 family members who had accessed and viewed their electronic health records participated in the sessions.

**Results:**

Four themes characterized patient experiences with reading the full complement of their health information. Patients felt that seeing their records positively affected communication with providers and the health system, enhanced knowledge of their health and improved self-care, and allowed for greater participation in the quality of their care such as follow-up of abnormal test results or decision-making on when to seek care. While some patients felt that seeing previously undisclosed information, derogatory language, or inconsistencies in their notes caused challenges, they overwhelmingly felt that having more, rather than less, of their health record information provided benefits.

**Conclusions:**

Patients and their delegates had predominantly positive experiences with health record transparency and the open sharing of notes and test results. Viewing their records appears to empower patients and enhance their contributions to care, calling into question common provider concerns about the effect of full record access on patient well-being. While shared records may or may not impact overall clinic workload, it is likely to change providers’ work, necessitating new types of skills to communicate and partner with patients.

## Introduction

As younger generations embrace technology, one of the oldest tools in medicine, the doctor’s note, is in its infancy of reform

TW Feeley and KI Shine, Annals of Internal Medicine, 2011

Forty years have passed since Shenkin and Warner proposed that patients routinely be given “complete and unexpurgated copy of all medical records, both inpatient and outpatient” [1]. They forecast that record sharing would enhance patient autonomy, improve patient-physician relationships, and serve as an educational tool. In the ensuing years, the few practices that have opened notes and test results to patients have confirmed such predictions [2-4]. A recent study, referred to as *Open Notes*, provided patients at three large US health systems access to primary care notes online [5]; the great majority of patients reported greater understanding of their medical issues and recall of their treatment plans [6]. Despite this, complete sharing of health records with patients remains an uncommon and controversial practice.

US law established the right of every patient to review their medical records or request amendments [7]. Few people obtain copies of their records due to a lack of awareness of this option and a cumbersome process [8]. Yet many adults want full access to their records [9-11], believing access will help with self-care [12]. Several large health systems offer patients a personal health record (PHR) to securely retrieve test results, make appointments, refill medications, and email providers [13-15]. Patients using PHRs have been shown to be more engaged in their health and have greater satisfaction with care [16-17]. Further, PHRs may be associated with improved health outcomes [18-20].

However, PHRs do not typically provide full access to clinical notes and test results. Physician barriers to sharing clinical notes with patients have been described [6,11,21]. Reluctance stems from concerns about patient harm or confusion, burden on clinical work, and questioning of physician performance. While not all patients will choose to view their records, and health literacy is likely to affect information accessibility, patient support for full sharing of records continues to escalate [22-24].

At the US Department of Veterans Affairs (VA), the initial PHR prototype offered military veterans a “virtual window” into their health record. The My Health*e*Vet Pilot afforded a unique opportunity for patients to access their complete health records, including primary care and specialty notes, discharge summaries, and laboratory and imaging results. The purpose of this study was to understand, using qualitative methods, the experience of patients who read their records using a PHR. We sought to determine if veterans who accessed their health data and notes felt that such access had an impact on their care or their relationships with their provider(s), and if they believed that access was associated with any unintended consequences.

## Methods

### My Health*e*Vet Pilot Program

**Figure 1:**
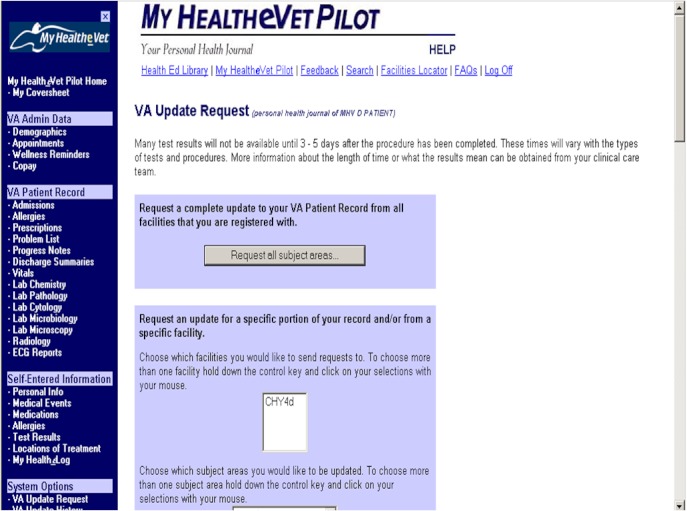
My Health*e*Vet Pilot landing page screenshot.

Between 2000 and 2010, nine VA facilities in Oregon, Florida, New York, and Washington, DC, recruited 7464 patients to enroll in the My Health*e*Vet Pilot. An enrolled patient completing in-person identity proofing could access clinic notes, hospital discharge notes, problem lists, vital signs, medications, allergies, appointments, and laboratory and imaging test results. Users could also manually enter personal data (eg, blood pressure, blood sugar, weight), access educational content, and authorize others to use the PHR on their behalf. Secure email with providers was not yet available through the PHR prototype. Figure 1 shows a screen shot of the portal landing page. Users could access their records until July 2010, after which time the Pilot was discontinued.

### Study Design and Setting

To explore patient perceptions of having full electronic access to their health records, we conducted a qualitative study using focus group interviews. A semistructured discussion guide (Table 1) was designed to elicit feedback about how participants accessed their information, whether and how communication with providers was affected, and emotional and behavioral experiences resulting from seeing their clinical information and providers’ notes. The study was approved by the Institutional Review Board of the Portland VA Medical Center.

### Sampling and Recruitment

This study was conducted at the Portland, Oregon VA Medical Center. This facility achieved the highest enrollment in the My Health*e*Vet Pilot program, with 72% (5361) of enrollees among nine Pilot sites. Since the primary goal was to interview patients with a recent experience viewing their health records, we chose to recruit those who accessed the PHR during the 18-month interval before recruitment. Portland VA patients were eligible if they logged in and accessed any part of their record between January 1, 2008, and June 30, 2009. Excluding patients who did not use the PHR or accessed records only before 2008, a total of 697 patients met the criteria for eligibility.

We used a purposive sampling strategy to recruit patients for 5 focus groups. For 4 groups, we targeted those who accessed their records 10 or more times during the 18-month interval, with the rationale that this level of use would ensure participants had gone beyond a trial of PHR use. For a fifth group, we recruited patients who accessed their record 2-3 times during the interval. This allowed us to examine the experiences of patients accessing their records less frequently.

Recruitment letters were mailed to a random sample of 126 eligible patients; 45 patients and 2 family delegates responded, of which 40 expressed interested in the study. The principal investigator telephoned the 40 responders, providing more information about the study and inviting each to attend a focus group. A total of 30 patients and 6 family members attended a session. Groups averaged 7 participants. Patient age ranged from 49-82 years and 4 patients (11%) were women. Five of the delegates were women. Participants signed informed consent for the study and for audio recording the session and were given US $25 reimbursement for travel.

### Data Analysis

Focus groups were conducted between November 2009 and January 2011 and moderated by an experienced facilitator (NP or SW). Interviews lasted between 60 and 90 minutes. Sessions were audio recorded and transcribed, then coded using a conventional content analysis approach in which themes of interest emerge inductively during analysis after all data are collected [25]. Two researchers independently coded the transcripts using QSR NVivo 9 software. The team met regularly to iteratively reach consensus on code definitions, identify themes, and resolve any coding discrepancies. Final intercoder agreement on individual codes ranged from 89% to 100%. Common broad themes identified included perceived benefits to self-care and perceived benefits to participation in care, as well as positive and negative experiences with the My Health*e*Vet Pilot program. More granular analysis of these themes is the focus of this paper.

**Table 1:** Focus group interview guide.

Item #	Questions
1.	Relate your experience looking at your medical records prior to the My Health*e*Vet Pilot.
2.	Talk about your experiences reading your medical records online and what type of information that you reviewed.
3.	How did you make sense of the medical records you viewed? What did you do when you read information that you didn’t understand?
4.	In your opinion, what has been positive about viewing your VA medical record?
5.	In viewing your records, was there anything that you didn’t anticipate or that surprised you?
6.	Have there been any negative issues with viewing the records? Have you experienced any stress or anxiousness from viewing any part of the records?
7.	Did you talk to your doctor or provider about viewing the record? Can you discuss a particular experience related to this?
8.	How has viewing your medical record impacted your relationship with your provider(s)? Have you noticed any changes in how your doctor or provider writes their notes?
9.	After seeing your medical records, did you request any changes to the content of the records?
10.	Did you find, as a result of viewing your records, that you changed (increased or decreased) the amount of times you called or visited the VA? If these changed, why do you think so?
11.	What are your feelings about continuing access to medical records in the future?
12.	Having been through the experience of having access to your medical records online, what would you want to tell others?
13.	What expectations did you have for My Health*e*Vet Pilot Program? What did you think or hope would happen, reading your medical records online?

## Results

Four broad themes characterized patients’ experiences with reading the full complement of their health information (summarized in Table 2). Three themes related to patients’ perceived benefits of electronic record access, and one theme focused on their concerns. First, patients reported that seeing their records had a positive effect on care communication between visits as well as during encounters. Second, access was felt to improve patients’ knowledge about their own health and prompted greater desire for self-care. Third, patients reported that health record access improved participation in their care in a variety of ways. Last, patients described challenges resulting from viewing clinical notes. Because there is a great deal of interest in the field regarding the potential for patient distress created by access to notes, our coding was highly sensitive to this category of comments. We analyzed the minor theme of patient difficulty in order to contribute to this discussion. Each theme is explored further, with examples of patients’ statements illustrating subthemes.

The analysis did not find any recurring thematic differences between the experiences of patients having higher frequency of PHR usage (Groups 2-5) compared to lower usage (Group 1). We analyzed the frequency of code occurrences, or how often a passage was assigned a particular code, and the coverage of codes, or percentage of total text to which a code was applied. For all groups, the thematic content was found to be similar. While there were more numerous, less detailed utterances by patients in Group 1, the perceived experiences of full record access were broadly shared across the focus groups. Therefore, our findings are pertinent to all focus groups.

### Perceived Enhanced Communication With Providers and Health Care Team

One benefit frequently described by patients was that access to health record information served to facilitate communication about their care. Patients reported better recall of appointments and care issues, felt more prepared for in-person visits, and found a greater ability to communicate with providers inside and outside the VA.

#### Communication Supplement

Access to the record was considered to be a valuable supplement to communicating in-person with providers. Several patients reported feeling less reliant on providers and staff to relay pertinent information during or between visits which, in turn, allowed them to avoid situations such as remembering in-person discussions or waiting for a phone call to be returned: “Then eVet came along and the write-ups were real great, and I didn’t have to wait 6 months to talk, or a year to talk to the doctor, find out what happened almost a year ago.” (FG3) and:

Often I get very stressed at a doctor’s appointment, don’t remember half of what’s going on and I could go on to eVet and get my information and go, “ok, we’re not in sync with this”…that helped a great deal

Focus Group Two, FG2

If they tell you something you don’t understand or you forget, because maybe it’s bad news or something, you go home and you really don’t remember. Somebody will say, “What did they tell you?” Well, I don’t know, but if you go on Health*e*Vet, you can find it.

FG4

#### Appointment Recall

A commonly cited benefit was assistance remembering appointments and scheduling follow-up. Patients felt shared records offered advantages over mailed letters that may get lost or misplaced: “I like the appointments. You know, sometimes you forget. You go in there every couple of days and ‘Oh yeah, I got one coming up’.” (FG4)

#### Preparing for Encounters

Access to notes was seen as a way to help prepare for clinic visits. Patients described how knowledge of record content allowed for a better understanding of what questions to ask and consequently, to improve the visit by leading to a more efficient encounter:

I can go in and ask more intelligent questions and we don’t have to spend as much time with them explaining everything to me. And then, with my stress level up at the doctor’s office, I don’t hear half of it and then we may have to do it again and again and so, it helps us to have better communication

FG5

It kind of better prepares me for the upcoming appointment, because I’ve got the data in my own hand. So, without starting out all over again, him repeating a whole lot of history, we can start a conversation at the treatment level we’re at right now.

FG5

#### Sharing Health Information

Patients described how electronic access to records helped them coordinate care between VA and nonVA providers. They focused on ease of access and how the information offered important data that otherwise might not have been readily available:

I got on it because I have a civilian doctor as well as VA. Because I live so far from VA, so if I have a problem I have a local doctor. It’s a hundred miles up here, so that’s the reason I want the records because he, my doctor, needs to see those records too.

FG1

### Perceived Improved Patient Knowledge and Self-Care

For many patients, access to their records increased perceived knowledge about their medical conditions and fostered a greater sense of control of their care. Several commented that seeing what was written about them prompted more efforts at self-care. Patients collectively and repeatedly discussed using the Internet to help understand medical information in their record.

#### Insight Into Health Conditions

Patients shared that access to clinical notes gave them what they perceived to be greater insight about their health conditions and treatment plans: “It was nice to be able to see what diagnoses you had, what your conditions were because sometimes, they’re just more than you can remember.” (FG2) and “Being a diabetic and having liver disease, it was a lot of information, a lot of instruction from doctors. I was always looking back to see what they said, instructions and everything.” (FG5)

#### Insight Into Provider Assessments

Patients also discussed experiencing benefit by gaining insight into their providers’ perspectives that came from reading clinical notes:

We talked about it when I was in there, but I don’t think I gathered it all up. To see it down in writing, I think helped me. He [the doctor] was concerned, and he put it down there. I think just seeing it in writing actually opened my eyes a little bit more.

FG2

Doctors aren’t real gabby and they never tell you everything. Even if you ask questions, they’ll sort of slide around them. They don’t have the time, you know. I found stuff out that I was just amazed at, truly, about myself.

FG4

#### Personal Control for Self-Care

Patients described wanting to be more responsible and take more control of their health issues as a direct result of reviewing their records, which was perceived as a positive influence:

It personally helped me assume the role of taking care of my own health, which my wife, a nurse, said, “I’m not taking care of you anymore. You’ve got to take care of you”. So, all of a sudden, I had access to the information so I could do that. That was very positive.

FG3

I found the cholesterol and all that other stuff, and it made me start thinking about my lifestyle and how I needed to do a little bit more on my own and not depend on the doctor to hand me pills and stuff. It encouraged me…I knew more. I understood more.

FG4

#### Self-Directed Internet Research

Patients in every group reported using online search tools to learn more about diagnoses, tests, and abbreviations. Patients felt that terminology could be confusing, yet they valued access to all the information and tried to understand medical jargon on their own before querying providers.

Well, you could just pop over to Google or go to the library in there, a dictionary in there, you could pop over and check it out and see what it’s saying instead of sitting there sweating it out trying to figure out what it is.

FG4

Well, sometimes I can figure out a problem myself either by my own online research or by just thinking about it and saying, “Well, I’m going to try this and fix it without the doctors involved.

FG2

### Perceived Greater Patient Contribution to Care

Patients brought their own perspectives about health care quality while talking about their experiences reading their records. Several patients shared how reading notes led to awareness of a service that was needed, or not needed, from their standpoint. They demonstrated how the information prompted more active patient participation in discussions about their health issues and their care.

#### Monitoring and Reminder Assistant

A number of patients described how access to their information, in their view, served as a way to monitor needed follow-up, ensuring that appropriate evaluation or treatment was completed: “I found out I was anemic. No one ever told me. Then when I asked the doctor about it, I was on iron pretty quick after that.” (FG4) and:

I had an ultrasound on my liver and I saw the results online. It said, “Re-do in six months”. Well, six months came around and nothing happened. So I called the doctor and say, “Well, it said here six months, re-do” and he said, “Well, let me look at your records”. He says, “Oh yeah, they did say that”. So, if I hadn’t reminded him, I probably wouldn’t have got it

FG5

#### Engaged to Discuss Health and Health Care

Patients talked about how reading their notes led to more dialogue with providers about what was written, including offering their opinion about whether or not the information was consistent with what had been discussed during visits:

My Oncologist was a pretty up-front guy. But I got on Health*e*Vet I found out he wasn’t as up front as I thought he was…with his comments, what he had written. So, when I went to see him the next time, I said, “I’d like to know, what you think and what you know, and what you’re predicting. So, rather than just write it in there, tell me and then write it”.

FG4

#### Participatory Care and Shared Decision-Making

Patients offered stories about how access to their records allowed them to play a more active role with their providers as an advocate about their care and treatment:

It just probably made me healthier than I might have been without having the information available, to either talk to the doctor, you know, just something as simple as changing a medication for something. You know, going, “Hey, look, the thing you got me on ain’t really working that great. Let’s try something different. What do you think about this?”

FG3

### Perceived Challenges From Reading Notes and Electronic Documentation

Patients were repeatedly probed about stress or harm related to reading notes. Three people responded directly that reading notes caused initial stress. In some instances, patients expressed discomfort about the language in notes, errors or inconsistencies in note content, or strain on patient-provider dialogue. At the same time, several patients voiced contrary views, opening up discussions about pros and cons of having their information available. A number of patients were frustrated by technical problems that became more prevalent prior to the closure of the Pilot.

#### Disclosure of Information

Statements illustrated challenges stemming from viewing newly revealed information that had not previously been disclosed to patients. One participant, a wife of a patient, expressed stress upon seeing an operative report; when asked if reading such notes was harmful, she denied harm had ensued: “I would rather not have known. There was a lot of little things they wrote, you know, step-by-step what had happened in his operation.” (FG1)

A second participant described concern about the potential for negative consequences from access to all of his health information:

I think that’s like a latent danger in all this information and having access to it. Meaning, I have access to my records and if there’s something that I don’t quite understand, I go to an outside source for feedback and even that might come across clear as mud. So now I’m sort of left with dis-information because it’s not serving me. That’s one of the dangers I see for me personally.

FG1

A few patients shed light on the challenge that greater information access provides data that are both helpful as well as worrisome: “I think being able to see the tests results has created, sometimes a little more stress because ‘Uh-oh, look at this.’ But overall, less worry, less stress because now I have facts.” (FG5) and:

Last year I had some mental health issues. I was able to see some things that I didn’t like what they said, one of them being about causing my own illness. Well, I read more about it online because I’m going, what does he actually mean by that? It explained a lot to me and calmed me down a lot. It really helped having the records and reading what they said.

FG2

Other participants denied stressful experiences from access to their information, feeling that having all of the health record available offered greater benefit than having partial information:

I think this is a way to encourage them to write the whole truth and all of the truth, you know, to say it all the way they feel it. The better for the patient. “Well, we don’t want to tell the person this because it may make them upset”…I say that’s a lot of bull. I want to know.

FG2

Just knowing....is better than not knowing, I think, in most things in life. Because you can imagine a lot of stuff in your health world, when you think, “Is this a pimple or am I dying of cancer?” You go through that whole thing. So, just knowing, just being able to review that and say, “Okay, I’m not dying of cancer. That’s a pimple”…gave me peace of mind.

FG3

One patient reported that their provider did not agree with this level of patient access: “My doctor, she didn’t like the fact that I could see the progress notes, saying there should always be a place where the doctors can write private notes to each other.” (FG4)

#### Language in Documentation

A small number of patients mentioned what they believed to be offensive language in notes. Some reported talking to the note writers about their observations:

There were a couple of cases where someone had written something derogatory about me. I was able to see it and find out the person fabricated what they were saying and I could bring that to the doctor’s and admin’s attention without having to plead my case. It was written in black and white.

FG5

I went to optometry, and the guy said, “These [glasses] aren’t VA, I can’t deal with them.” Well, I left. Then I was looking through my notes on something else, and I ran across this guy. And “hostile” was the word he used [in the note]. I felt there was a wall there after that.

FG1

#### Inconsistencies in Content

Several patients perceived inconsistencies in notes, citing that information given verbally at visits was not equivalent to that written in notes. Some patients spoke with providers about these discrepancies; many did not. A few formally requested a change in the record. One patient discussed seeking an alternate provider:

One time I found something totally wrong in his notes. It was like not even me. When I told the doctor, he corrected it. I went back in after the visit and read it, and he had, he’d gone in and edited his notes. It was a real mistake, which could have cost me down the road.

FG3

And sometimes, you want to change doctors as a result of what the doctor said. Not because he’s describing it wrong, but he’s describing it incompetently because you know yourself better than he does in many cases

FG2

#### Observations on Electronic Records

Several patients commented about electronic records and the ability to access information remotely. Some expressed a preference for notes that were thorough and without redundancies; one man criticized the presence of “boilerplate” elements in notes. A few patients voiced frustration over technical issues with the Pilot, particularly in 2010 when notes were not updating in a timely manner. All patients were disappointed with its impending closure:

But the Pilot is part of the whole system and if we can make things better by using the pilot and making our suggestions, it makes it better for all the veterans who’ve had bad experiences and that’s a good start for the VA.

FG2

Communication is the key and if you can’t communicate with your physician, either electronically or verbally, and this particular vehicle that we had given to us, was to me personally one of the best communication tools that the VA’s ever come out with.

FG3

**Table 2:** Summary of themes on patient experiences with full record access.

**Theme 1: Perceived enhanced communication with providers and health care teams**	
	Supplements in-person communication
	Improved recall of appointments
	More prepared for encounters with providers
	Greater ability to share information with non-VA providers
**Theme 2: Perceived improved patient knowledge and self-care**	
	Improved understanding of health issues
	Greater insight into provider assessments and recommendations
	Improved sense of control of health issues
	Prompt to use the Internet to understand information
**Theme 3: Perceived greater patient participation in care**	
	Prompt to remind health care team for appropriate care or follow-up
	More engaged to discuss health and health care issues
	More able to participate in decisions if care is needed or not
**Theme 4: Perceived challenges from viewing records and electronic documentation**	
	Stress related to information not routinely disclosed
	Concern about language in notes
	Inconsistencies or errors in documentation
	Observations on electronic records and PHR technical problems

## Discussion

Our findings support prior qualitative research that shows full health record access is empowering for patients and caregivers [26]. Patients’ perspectives provide insight into how shared notes can foster active patient participation in their care. In all focus groups, participants put knowledge from their records to use by learning more about their health issues, gaining more knowledge about their providers’ views, and advocating for themselves in discussions about their care. Reading health information in an unpressured manner allowed patients time to contemplate its content and meaning. Records were also a starting place for online research. As a result, patients felt more prepared for clinic visits but sometimes were also less likely to call the clinic or request an appointment. Of particular interest were stories of patients serving as their own “clinical reminders”, making an effort to improve the quality of their care by ensuring follow-up care was provided.

While participants reported that viewing their records was positive, a number described some difficulty upon seeing clinical notes. Predominant issues identified were the use of derogatory terms, stress that initially emerged from reading detailed personal information, and challenging conversations with members of their health care teams. At the same time, most participants, including many who cited these concerns, believed it was important and valuable to have all of their health record data. Of note, study participants viewing their records a few times expressed similar themes to those logging in more frequently, suggesting that patient use of PHRs at any given time is driven by dynamic factors such as personal health needs, rather than by initial positive or negative experiences accessing records.

While our study did not identify appreciable harm, small but significant concerns about negative consequences of sharing records remain. In some instances, patient-provider communications about shared records created discomfort. It appears that all parties needed to adjust to a new dynamic, with patients having higher expectations of disclosure of information. Such issues were described in the *Open Notes* study [6], pointing to a need for professional education on clinical documentation and communication that optimizes patient participation and shared decision-making.

Some limitations of the study must be noted. Patients were recruited from a single medical center and viewed their records during a specific time interval, and therefore may not be generalizable to the VA patient population or My Health*e*Vet Pilot users overall. Study responders could have been more satisfied with the PHR or had more positive experiences overall with reading their health records. Second, many patients enrolled in the Pilot program yet did not view their records during the 18 months before the study, so they were not eligible for recruitment. Pilot enrollment occurred early in the 10 years it was active, and the study was conducted towards the end of this period. There were likely many patients who enrolled but chose not to access their records, had technical problems using the site, or viewed their records years before but did not to do so again. Further, release of the national My Health*e*Vet portal in 2003 likely caused Pilot enrollees to stop using the PHR prototype. Since our goal was to learn from patients who were able to recall using the Pilot, it was important to recruit those who accessed their health records relatively recently.

### Study Implications

To our knowledge, this is the first qualitative study of veteran patients’ experience viewing electronic records that included clinical notes and test results. Our findings have important implications for the development of electronic health records and PHRs. While patients by and large welcome full record access, clinicians reveal protective postures and worry about patient distress and confusion, resulting in more work for staff [19,21]. Our study, focusing on the patient experience, suggests that the first two of these expectations are overestimated. Concern about workload is likely more complex. Patients’ accounts suggest that sharing all records reduce workload in some areas, for example, fewer visits or decreasing requests for copies of records. At the same time, participants’ experiences also challenge traditional roles for patients and physicians. While patient-provider conversations may prove uncomfortable, they also demonstrate greater patient participation in care and contribution to care delivery. As evidence shows that activated patients achieve higher levels of self-care and satisfaction [27], sharing all clinical notes with patients and their delegates could serve as a fundamental component for the meaningful use of electronic records and health information exchange.

In this era of greater transparency and technology designed to optimize the user experience, new skills will be needed to achieve shared care planning and decision-making. Ultimately, patient access to all health record notes may translate into care that is more effective and more satisfying—for both patients and for health professionals.

## Abbreviations

PHR: personal health record
VA: US Department of Veterans Affairs

## References

[1] Shenkin BN, Warner DC (1973). Sounding board. Giving the patient his medical record: a proposal to improve the system. N Engl J Med.

[2] Baldry M, Cheal C, Fisher B, Gillett M, Huet V (1986). Giving patients their own records in general practice: experience of patients and staff. Br Med J (Clin Res Ed).

[3] Fischbach RL, Sionelo-Bayog A, Needle A, Delbanco TL (1980). The patient and practitioner as co-authors of the medical record. Patient Couns Health Educ.

[4] Fisher B, Britten N (1993). Patient access to records: expectations of hospital doctors and experiences of cancer patients. Br J Gen Pract.

[5] Delbanco T, Walker J, Darer JD, Elmore JG, Feldman HJ, Leveille SG, Ralston JD, Ross SE, Vodicka E, Weber VD (2010). Open notes: doctors and patients signing on. Ann Intern Med.

[6] Delbanco T, Walker J, Bell SK, Darer JD, Elmore JG, Farag N, Feldman HJ, Mejilla R, Ngo L, Ralston JD, Ross SE, Trivedi N, Vodicka E, Leveille SG (2012). Inviting patients to read their doctors' notes: a quasi-experimental study and a look ahead. Ann Intern Med.

[7] Office for Civil Rights, HSS (1996). Public Law, 104-191.

[8] Fowles JB, Kind AC, Craft C, Kind EA, Mandel JL, Adlis S (2004). Patients' interest in reading their medical record: relation with clinical and sociodemographic characteristics and patients' approach to health care. Arch Intern Med.

[9] Hassol A, Walker JM, Kidder D, Rokita K, Young D, Pierdon S, Deitz D, Kuck S, Ortiz E (2004). Patient experiences and attitudes about access to a patient electronic health care record and linked web messaging. J Am Med Inform Assoc.

[10] Walker J, Ahern DK, Le LX, Delbanco T (2009). Insights for internists: "I want the computer to know who I am". J Gen Intern Med.

[11] Ross SE, Todd J, Moore LA, Beaty BL, Wittevrongel L, Lin CT (2005). Expectations of patients and physicians regarding patient-accessible medical records. J Med Internet Res.

[12] Winkelman WJ, Leonard KJ, Rossos PG (2005). Patient-perceived usefulness of online electronic medical records: employing grounded theory in the development of information and communication technologies for use by patients living with chronic illness. J Am Med Inform Assoc.

[13] Ralston JD, Martin DP, Anderson ML, Fishman PA, Conrad DA, Larson EB, Grembowski D (2009). Group health cooperative's transformation toward patient-centered access. Med Care Res Rev.

[14] Liang L, Kaiser (2010). Connected for health: using electronic health records to transform care delivery.

[15] Reti SR, Feldman HJ, Ross SE, Safran C (2010). Improving personal health records for patient-centered care. J Am Med Inform Assoc.

[16] Ralston JD, Hirsch IB, Hoath J, Mullen M, Cheadle A, Goldberg HI (2009). Web-based collaborative care for type 2 diabetes: a pilot randomized trial. Diabetes Care.

[17] Nazi KM (2010). Veterans' voices: use of the American Customer Satisfaction Index (ACSI) Survey to identify My Health*e*Vet personal health record users' characteristics, needs, and preferences. J Am Med Inform Assoc.

[18] Zhou YY, Garrido T, Chin HL, Wiesenthal AM, Liang LL (2007). Patient access to an electronic health record with secure messaging: impact on primary care utilization. Am J Manag Care.

[19] Earnest MA, Ross SE, Wittevrongel L, Moore LA, Lin CT (2004). Use of a patient-accessible electronic medical record in a practice for congestive heart failure: patient and physician experiences. J Am Med Inform Assoc.

[20] Green BB, Cook AJ, Ralston JD, Fishman PA, Catz SL, Carlson J, Carrell D, Tyll L, Larson EB, Thompson RS (2008). Effectiveness of home blood pressure monitoring, Web communication, and pharmacist care on hypertension control: a randomized controlled trial. JAMA.

[21] Walker J, Leveille SG, Ngo L, Vodicka E, Darer JD, Dhanireddy S, Elmore JG, Feldman HJ, Lichtenfeld MJ, Oster N, Ralston JD, Ross SE, Delbanco T (2011). Inviting patients to read their doctors' notes: patients and doctors look ahead: patient and physician surveys. Ann Intern Med.

[22] Bhavnani V, Fisher B, Winfield M, Seed P (2011). How patients use access to their electronic GP record--a quantitative study. Fam Pract.

[23] Greenhalgh T, Wood GW, Bratan T, Stramer K, Hinder S (2008). Patients' attitudes to the summary care record and HealthSpace: qualitative study. BMJ.

[24] Zulman DM, Nazi KM, Turvey CL, Wagner TH, Woods SS, An LC (2011). Patient interest in sharing personal health record information: a web-based survey. Ann Intern Med.

[25] Hsieh HF, Shannon SE (2005). Three approaches to qualitative content analysis. Qual Health Res.

[26] Fisher B, Bhavnani V, Winfield M (2009). How patients use access to their full health records: a qualitative study of patients in general practice. J R Soc Med.

[27] Greene J, Hibbard JH (2012). Why does patient activation matter? An examination of the relationships between patient activation and health-related outcomes. J Gen Intern Med.

